# Acupuncture for fecal incontinence: Protocol for a systematic review and data mining

**DOI:** 10.1097/MD.0000000000014482

**Published:** 2019-02-15

**Authors:** Haixiong Lin, Zhiqing Zhang, Guijuan Hu, Xiaotong Wang, Chunni Lin, Yongjun Chen

**Affiliations:** a The First School of Clinical Medicine; b South China Research Center for Acupuncture and Moxibustion; c Medical School of Acu-Moxi and Rehabilitation, Guangzhou University of Chinese Medicine, Guangzhou; d School of Foreign Languages, Xinhua College of Sun Yat-sen University, Dongguan, People's Republic of China.

**Keywords:** acupuncture, complementary and alternative therapy, electroacupuncture, fecal incontinence, laser acupuncture, protocol, systematic review

## Abstract

**Background:**

Fecal incontinence is a socially and emotionally destructive condition that has a negative impact on personal image, self-confidence, and quality of life. Acupuncture is commonly used to treat chronic conditions, including fecal incontinence. However, no relevant systematic review or meta-analysis has been designed to evaluate the effects of acupuncture on fecal incontinence.

**Methods:**

We will identify relevant randomized controlled trials (RCTs) from the Cochrane Library, Medline, Embase, PubMed, Springer, Web of Science, China National Knowledge Infrastructure, VIP Chinese Science and Technology Journals Database, Wanfang database, and clinical trial registration center from their inception to February 28, 2019. The primary outcome measures will be clinical effective rate, functional outcomes, and quality of life. Data that meets the inclusion criteria will be extracted and analyzed using RevMan V.5.3 software. Two reviewers will evaluate the studies using the Cochrane Collaboration risk of bias tool. Publication bias will be assessed by funnel plots, Egger test, and Begg test using the Stata software. Acupoints characteristics will be analyzed by Traditional Chinese Medicine inheritance support system.

**Results:**

This study will analyze the clinical effective rate, functional outcomes, quality of life, daily average number of fecal incontinence, and effective prescriptions of acupuncture for patients with fecal incontinence.

**Conclusion:**

Our findings will provide evidence for the effectiveness and potential treatment prescriptions of acupuncture for patients with fecal incontinence.

**PROSPERO registration number:**

PROSPERO CRD42019119680.

## 1 Introduction

Fecal incontinence is characterized by the impaired ability to control stool or gas to allow evacuation at a socially acceptable time and location,^[1]^ affecting approximately 7% to 15% of women in the community, causing social stigma, embarrassment, and impaired hygiene.^[2]^ It has been confirmed in numerous studies that fecal incontinence affects personal image and self-confidence, and even leads to social isolation, resulting in a significant impact on quality of life.^[3,4]^ Previous studies have also shown that obstetric trauma and irritable bowel syndrome are common risk factors for fecal incontinence.^[5,6]^ Currently, management or treatment measures for fecal incontinence include bowel routines, changing dietary habits, biofeedback training, electrostimulation, and surgical interventions, as well as supportive care measures such as pads, deodorants, and protective ointments.^[7,8]^ However, only 46% of patients who underwent surgery for prolapse reported continence improvement at 38.9 months of follow-up, while those who underwent an operative procedure other than ventral rectopexy were similar to nonoperated patients.^[9]^ Therefore, a growing number of people are seeking conservative therapies, including dietary changes, antidiarrheal medications, bulking agents, enemas, and biofeedback,^[10,11]^ as well as complementary and alternative therapy.^[12]^

As a kind of complementary and alternative therapy, acupuncture is often used to treat chronic functional diseases, and its application in fecal incontinence has gradually spread in recent years.^[13]^ Compared with sham acupuncture, acupuncture at the lumbosacral region is effective for women suffering from stress urinary incontinence^[14]^; the similar but slightly different clinical outcomes can be seen in nerve stimulation,^[15]^ which may also have an impact on functional outcomes or quality of life of patients with fecal incontinence. In recent years, the number of clinical trials involving acupuncture treatment of fecal incontinence has increased significantly^[16,17]^; however, the clinical efficacy and potential treatment prescriptions of acupuncture for fecal incontinence remain unclear, prompting us to further explore its efficacy and effective prescription. In this study, we will investigate current evidence associated with the effectiveness and safety of acupuncture for fecal incontinence, which will help clinicians use it in clinical practice better.

## 2 Methods

### 2.1 Study type

We will collect articles of randomized controlled trials (RCTs) that evaluated the clinical effective rate, functional outcomes, quality of life, daily average number of fecal incontinence, or side effects of acupuncture on fecal incontinence for systematic review and meta-analysis. RCTs, observational studies, case series, and case–control studies will be included for data mining. Observational studies, case series, case–control studies, animal experiments, qualitative studies, proceedings, conferences, comments, and review articles will be excluded in the meta-analysis. There will be no restrictions regarding the race, region of the studies, gender, and age of patients.

### 2.2 Participants

Participants who are diagnosed with fecal incontinence will be included. Fecal incontinence should be confirmed according to the 1999 consensus standard of the American Association of Colorectal Surgeons.^[18]^ Participants with urinary and fecal incontinence will be excluded.

### 2.3 Interventions

Participants in the intervention group are those undergoing acupuncture, including electroacupuncture therapy, acupuncture alone, laser acupuncture, and warm acupuncture. There will not be any restrictions on age and original countries of the participants. Control group intervention could be no treatment, sham or placebo acupuncture, electrical stimulation, etc. The other treatments between intervention group and control group should be the same.

### 2.4 Outcome measures

The primary outcomes will include clinical effective rate, functional outcomes, quality of life. Functional outcomes will be calculated on the basis of the Wexner score.^[19]^ The criteria of quality of life according to the Fecal Incontinence Quality of Life Scale was developed by Rockwood et al,^[4]^ including thoughts about lifestyle, depression/self-perception, coping/behavior, and embarrassment. The Fecal Incontinence Quality of Life Scale measures impairment caused by fecal incontinence or fear of fecal incontinence, ranging from 1 (most of the time) to 4 (none of the time).^[5]^

The secondary outcomes will be daily average number of fecal incontinence, adverse events, and discontinuations due to adverse events.

### 2.5 Search strategy

An electronic search will be conducted. We will identify relevant studies from The Cochrane Library, Medline (Ovid), Embase, PubMed, Springer, Web of Science, China National Knowledge Infrastructure, VIP Chinese Science and Technology Journals Database, Wanfang database from their inception to February 28, 2019. The search term will consist of 3 parts: intervention method, disease, and study type: (“electroacupuncture therapy” or “acupuncture” or “laser acupuncture” or “warm acupuncture” or “electroacupuncture” or “acupuncture therapy” or “laser acupuncture therapy” or “warm acupuncture therapy”) and (“fecal incontinence” or “FI” or “encopresis” or “defecation incontinence”) and (“randomized controlled trial” or “randomized” or “case control studies” or “observational studies” or “case series” or “trial”) and (“blind”). The details of the PubMed and Wanfang database search strategies are provided in Tables 1 and 2. The similar but adaptive search strategies will be applied to other electronic databases. Language will be restricted to English and Chinese. Reference lists of relevant original studies will be screened to identify additional potentially citations. In addition, the following 3 trial registries will be searched for ongoing studies: Current Controlled Trials: www.controlled-trials.com; Clinical Trials: www.ClinicalTrials.gov; and Chinese Clinical Trial Registry: www.chictr.org.cn/index.aspx.

**Table 1:**
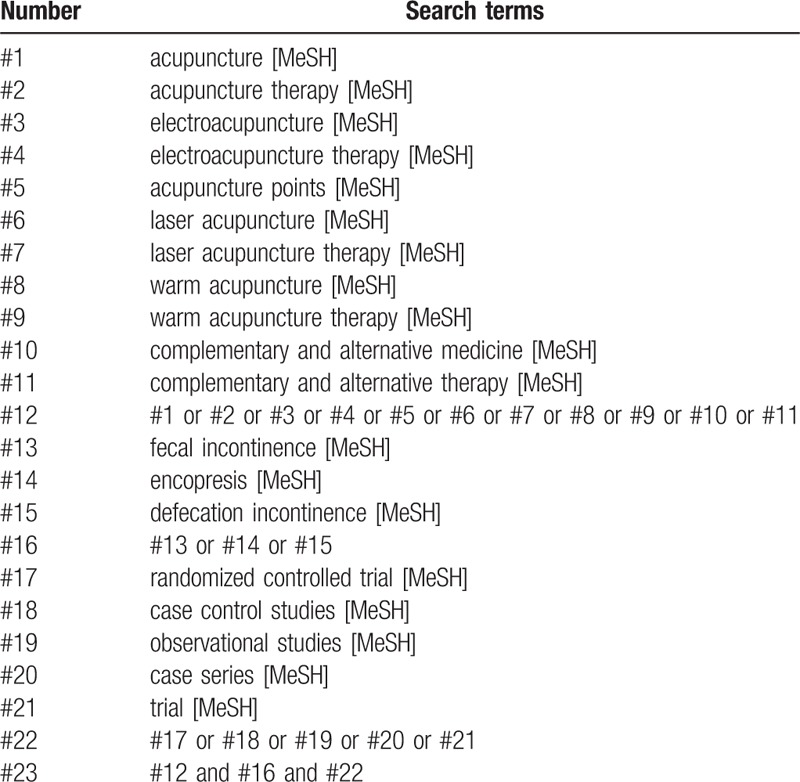
The search strategy for PubMed database.

**Table 2:**
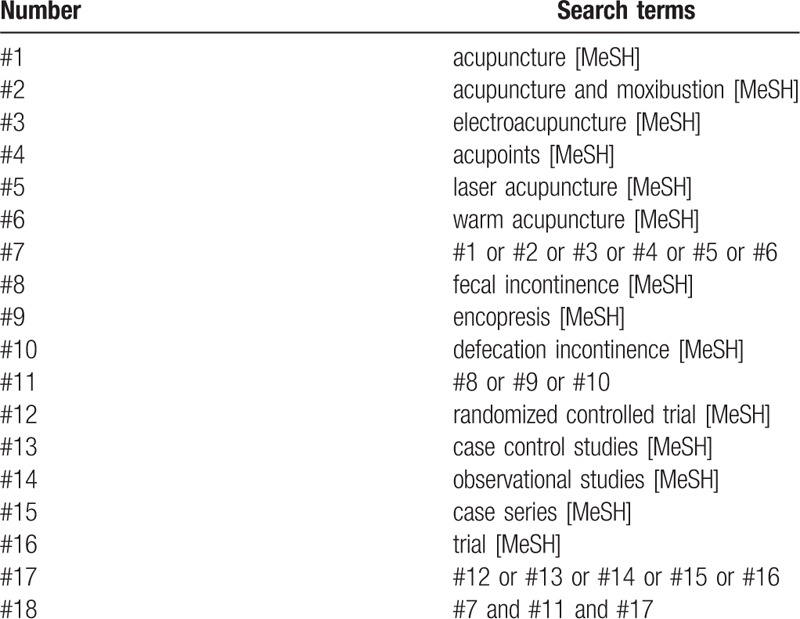
The search strategy for Wanfang database.

### 2.6 Study selection and data extraction

**Figure 1:**
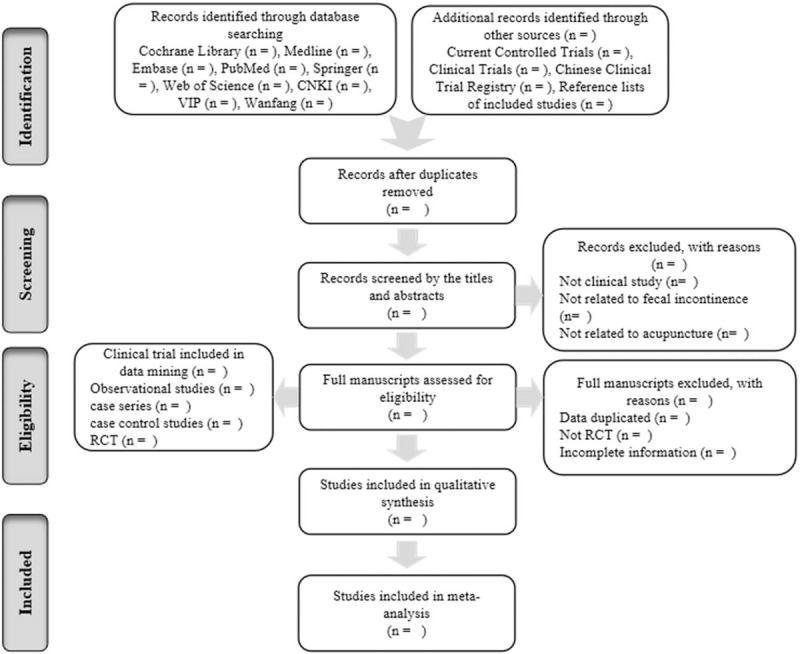
Flow chart of the search process.

Author (Chen YJ) with experience in the field will guide the search. All retrieved results will be imported into NoteExpress 3.2.0 (Available at: http://www.inoteexpress.com/aegean/). Duplicate citations from different databases will be identified and excluded. Two reviewers (Lin HX and Hu GJ) will independently screen the titles and abstracts of citations to delete irrelevant studies, and then examine the full-text reports, identify potential studies, and assess eligibility of studies for inclusion. Any disagreement will be resolved on the eligibility of studies through discussion and consensus or, if necessary, with the help of a third partner (Chen YJ). We will record details of the excluded studies and the reasons for exclusion. The selection process will be showed in a PRISMA flow chart (http://www.prisma-statement.org/) (Fig. 1). Two review authors (Lin HX, Hu GJ) will extract data using a data extraction form according to the recommendations of the Cochrane Handbook for Systematic Reviews of Interventions. The author's name, publication year, sample sizes, age of participants, gender, treatment method, dropout number, clinical outcome, and follow-up periods will be extracted. Disagreements will be resolved by consensus or through discussion with a third review author (Chen YJ).

### 2.7 Addressing missing data or unclear measurement scales

We will obtain the missing data or additional information by contacting the study authors via email or telephone if possible. Otherwise, we will analyze the available information and conduct sensitivity analysis to explore the potential impact of insufficient information on the results of the meta-analysis.

### 2.8 Risk of bias in included studies

Two review authors (Lin HX, Hu GJ) will independently evaluate each included study and will follow the domain-based evaluation as developed by the Cochrane Handbook for Systematic Reviews of Interventions. They will assess the following domains: Randomization sequence generation, allocation concealment, blinding of outcome assessors, incomplete outcome data, selective outcome reporting, other sources of bias. Each domain will be divided into 3 categories: low, unclear, and high.

### 2.9 Data synthesis and analysis

Effect sizes with their 95% confidence intervals (95% CIs) will be extracted. We will analyze the data using RevMan software (Version 5.3) (Available at: https://community.cochrane.org/help/tools-and-software/revman-5) provided by The Cochrane Collaboration.^[20]^ A meta-analysis using random or fixed effects models will be conducted to summarize the data. Continuous data will be pooled and presented as mean differences or standardized mean difference with their 95% CI. Dichotomous data will be pooled and expressed as risk ratio with their 95% CI. Statistical heterogeneity will be investigated using the Chi-square test and *I*^*2*^ values.^[21]^ We will interpret it using the following criteria: *I*^*2*^ values of 25% are considered low levels of heterogeneity, 50% indicated moderate levels, and 75% indicated high levels.^[22]^ As low or moderate heterogeneity suggests little variability among these studies, the data will be analyzed in a fixed-effects model.^[23]^ When significant heterogeneity occurs among the studies (*P* < .05, *I*^*2*^ > 50%), a random-effect model will be performed to analyze the data,^[24]^ as well as meta-regression will be adopted to investigate the potential sources of heterogeneity.

### 2.10 Additional analyses

Subgroup analysis will be conducted to evaluate the specific influence of intervention type, study quality, study location, treatment duration on pooled results. If the data are insufficient, qualitative synthesis will be conducted instead of quantitative synthesis. In addition, sensitivity analysis will be performed to examine the robustness of the results by eliminating low quality trials. We will also use Traditional Chinese Medicine Inheritance Support System to analyze the acupoints characteristics of acupuncture for fecal incontinence.

### 2.11 Assessment of reporting biases

Reporting bias will be evaluated by visual inspection of Funnel plots. At the same time, Begg test and Egger test will be used to test whether the funnel plot is symmetrical. A *P* value < .05 in Egger test or Begg test is considered statistically significant.

### 2.12 Confidence in cumulative evidence

In order to better prepare results for usage in guideline development, the quality of evidence for different outcomes will be graded according to the Grading of Recommendations Assessment, Development and Evaluation (GRADE).^[25]^ GRADE will be used to summarize the limitations in design, consistency, directness, precision, publication bias. The quality of each evidence will be divided into 4 levels: high, medium, low, and very low. Disagreements will be resolved by consensus.

## 3 Discussion

Fecal incontinence is an embarrassing condition involving unintentional loss of solid or liquid feces.^[26]^ It is a socially and emotionally destructive disease that has a negative impact on quality of life of suffers.^[27]^ In the United States, the 4-year incidence of fecal incontinence among the community residents over 65 years old is 17%.^[28]^ And the prevalence increases with age, affecting 16.16% of adults over the age of 70.^[29]^ At the same time, previous studies have confirmed that advancing age, pelvic floor dysfunction, parity, stool form disturbance, urinary incontinence, and multiple chronic diseases are potential pathophysiological mechanisms of fecal incontinence.^[30–32]^ At present, the treatment of fecal incontinence vary from medical methods, including biofeedback to various surgical interventions, but the results are still unsatisfactory.^[33,34]^ A number of patients with fecal incontinence attempt to use complementary and alternative therapy, including acupuncture.^[34]^

Adequate anorectal sensation combined with pelvic floor muscle strength helps maintain fecal continence.^[33,35]^ It is confirmed that acupuncture could relieve pain and regulate peripheral circulation, which is beneficial for bladder dysfunction and even gastrointestinal dysfunction.^[36]^ Besides, electroacupuncture stimulation combined with bone marrow mesenchymal stem cell transplantation can effectively repair the impaired anal sphincters.^[37]^ Therefore, acupuncture is gradually being applied to the treatment of fecal incontinence.^[13]^ To the best of our knowledge, even though acupuncture is often used for fecal incontinence, there is no planned or published systematic review of the effectiveness and safety of acupuncture for fecal incontinence. The purpose of this study is to evaluate the effect of acupuncture on clinical effective rate, functional outcomes, quality of life, daily average number of fecal incontinence, adverse events, and discontinuations events in patients with fecal incontinence. In particular, we will analyze specific acupuncture prescriptions that are used in fecal incontinence by Traditional Chinese Medicine Inheritance Support System. Herein, this study will be the first to assess the clinical efficacy and effective prescriptions of acupuncture for patients with fecal incontinence, and may benefit practitioners in the fields of complementary and alternative therapies.

## 4 Ethics and dissemination

Ethics approval is not required due to this work is carried out on published data. We aimed to explore the clinical effective rate, functional outcomes, quality of life, daily average number of fecal incontinence, as well as effective prescriptions of acupuncture for patients with fecal incontinence. In the end, the results will be submitted to a peer-reviewed journal.

## Author contributions

Lin HX and Wang XT had the original idea of this work and drafted the protocol. Hu GJ and Zhang ZQ designed the search strategies. Chen YJ proposed some advice for the design and revision. Lin CN designed the flow chart. All authors critically revised the draft and approved the final manuscript.

**Conceptualization:** Haixiong Lin, Xiaotong Wang.

**Data curation:** Zhiqing Zhang, Guijuan Hu, Xiaotong Wang.

**Formal analysis:** Haixiong Lin, Xiaotong Wang.

**Funding acquisition:** Yongjun Chen.

**Methodology:** Guijuan Hu, Chunni Lin.

**Project administration:** Xiaotong Wang, Yongjun Chen.

**Resources:** Haixiong Lin, Zhiqing Zhang, Guijuan Hu, Xiaotong Wang.

**Software:** Xiaotong Wang, Chunni Lin.

**Supervision:** Yongjun Chen.

**Validation:** Haixiong Lin, Xiaotong Wang.

**Visualization:** Yongjun Chen.

**Writing – original draft:** Haixiong Lin, Xiaotong Wang.

**Writing – review & editing:** Zhiqing Zhang, Guijuan Hu, Yongjun Chen.

Haixiong Lin orcid: 0000-0002-9939-7698.
